# Matrix protein 1 (M1) of influenza A virus: structural and functional insights

**DOI:** 10.1080/22221751.2025.2558881

**Published:** 2025-09-09

**Authors:** Yanting Zhu, Yixue Sun, Xiaoyu Deng, Peige Cao, Siqi Li, Huilin Yu, Shouzhi Sheng, Yanlong Cong

**Affiliations:** a State Key Laboratory for Diagnosis and Treatment of Severe Zoonotic Infectious Diseases, Key Laboratory for Zoonosis Research of the Ministry of Education, and College of Veterinary Medicine, Jilin University, Changchun, People’s Republic of China; b Jilin Research & Development Center of Biomedical Engineering, Changchun University, Changchun, People’s Republic of China; c Institute of Special Animal and Plant Sciences, Key Laboratory of Special Animal Epidemic Disease of the Ministry of Agriculture, Chinese Academy of Agricultural Sciences, Changchun, People’s Republic of China; d College of Plant Science, Jilin University, Changchun, People’s Republic of China

**Keywords:** Influenza A virus, matrix ptotein 1, structure, viral lifecycle, antiviral target

## Abstract

Enveloped viruses rely on matrix proteins for structural integrity and lifecycle progression. Matrix protein 1 (M1) is the most abundant structural protein of influenza A virus (IAV), playing a multifaceted role in viral uncoating, polymerase activity, vRNA transcription and replication, and assembly and budding. The M1 protein not only interacts with host cells but also regulates viral morphogenesis, thereby influencing viral transmissibility and pathogenicity. These properties make it a highly promising antiviral target. This review elucidates the intricate structure and function of M1, highlighting its regulatory influence on IAV replication and adaptation, and providing critical insights into viral replication dynamics and epidemiological shifts. Beyond engaging host factors, the M1 protein orchestrates virion morphogenesis – thereby shaping viral transmissibility and pathogenicity-making it a highly attractive antiviral target.

**Glossary**
CA-04: A/California/4/2009 (H1N1)
CRM1: chromosome maintenance 1
CTD: C-terminal domain
ER: endoplasmic reticulum
HA: hemagglutinin
HDAC6: histone deacetylase 6
IAV: influenza A virus
M1: matrix protein 1
M2: matrix protein 2
MTOC: microtubule-organizing centre
NA: neuraminidase
NEP: nuclear export protein
NES: nuclear export signal
NL602: A/Netherlands/602/2009 (H1N1)
NLS: nuclear localization signal
NP: nucleoprotein
NS1: non-structural protein 1
NS2: non-structural protein 2
NTD: N-terminal domain
PA: polymerase acidic protein
PB1: polymerase basic protein 1
PB2: polymerase basic protein 2
PM: plasma membrane
PR8: A/Puerto Rico/8/1934 (H1N1)
RdRp: RNA-dependent RNA polymerase
TNPO1: nuclear import factor transportin 1
VLPs: virus-like particles
vRNPs: viral ribonucleoproteins
WSN: A/WSN/1933 (H1N1)

Matrix proteins (M) are essential structural components in many enveloped viruses. They act as a bridge between the lipid envelope and the inner capsid, providing structural stability to virus particles and playing a critical role in regulating key life processes including virus assembly, budding, and host cell invasion [1–4]. Although M vary in sequence and additional functions across different viruses, their fundamental roles in maintaining virus particle integrity and promoting efficient replication are consistent. Given this understanding of the universal function of M, the influenza virus serves as a well-studied example that clearly illustrates the multiple functions of M in the viral life cycle. This paper systematically reviews the structural characteristics and biological functions of the influenza virus M1 protein, with a particular focus on its role in key stages of the viral lifecycle, including viral uncoating, genome transcription and replication, viral assembly and budding. By examining the M1 protein's functions in viral assembly and release, this review aims to provide a theoretical background and reference for considering M1 as a potential antiviral target, offering insights that could inform the development of novel strategies to combat influenza virus infections in the future.

## Influenza A virus genome and protein functions

Influenza A virus (IAV), a member of the genus *Alphainfluenza* within the family *Orthomyxoviridae*, is renowned for its ability to infect a broad spectrum of avian and mammal species. The IAV genome comprises eight segments of negative-sense, single-stranded RNA, which confer high genetic flexibility and encode at least 22 proteins [5]. Segments 1–3 encode polymerase basic protein 2 (PB2), polymerase basic protein 1 (PB1), and polymerase acidic protein (PA), which together form the viral RNA-dependent RNA polymerase (RdRp) complex that is essential for viral transcription and replication. Through leaky ribosomal scanning, segment 2 also produces PB1-N40, an N-terminally truncated isoform that modulates polymerase activity, and the accessory protein PB1-F2, which suppresses host antiviral responses by inducing mitochondrial apoptosis [5, 6]. The hemagglutinin (HA) protein, encoded by segment 4, is a viral surface glycoprotein that mediates binding to sialic acid receptors on host cells and subsequent membrane fusion. HA is a major target of neutralizing antibodies, and its antigenic variation forms the basis for influenza subtype classification. Segment 5 encodes the nucleoprotein (NP), which encapsulates vRNA and, together with the polymerase complex, constitutes the viral ribonucleoprotein (vRNP). NP is essential for maintaining genome stability, mediating nucleocytoplasmic trafficking, and regulating transcription and replication. Segment 6 encodes the neuraminidase (NA), a glycoprotein that cleaves sialic acids to enable the release and spread of progeny virions. Like HA, antigenic differences in NA contribute to subtype determination [7, 8]. Segment 7 encodes the matrix protein 1 (M1), the most abundant structural protein in IAV. M1 forms a matrix layer beneath the viral envelope, interacting externally with the cytoplasmic tails of transmembrane proteins HA and NA, and internally with the eight vRNPs, thereby maintaining the virion's integrity and fulfilling multiple functions across the viral life cycle [7, 9]. Segment 8 encodes the non-structural protein 1 (NS1), a major antagonist of host innate immunity that suppresses the interferon responses through multiple mechanisms. Because IAV transcription and replication occur within the host cell nucleus, the virus exploits the host splicing machinery to generate additional proteins, including matrix protein 2 (M2), matrix protein 42 (M42), non-structural protein 2 (NS2), and non-structural protein 3 (NS3). Among them, M2, also known as the ion channel protein that regulates endosomal pH and facilitates uncoating, and NS2, also termed nuclear export protein (NEP), which collaborates with M1 to mediate the nuclear export of progeny vRNPs [5, 6]. A summary of these proteins is provided in Table 1.

**Table 1.** Genomic characteristics and protein functions of influenza A virus [5, 6].

Segments	Proteins	Length (aa)	mRNAs	Reading frames	Functions
1	PB2	∼759	non-spliced mRNA	Translated from AUG1	Component of RNA polymerase complex; recognizes and cleaves the 5′ cap structure of host mRNA as primer
	PB2-S1	∼508	alternatively spliced mRNA	Translated from AUG1; yielding a 495 aa truncated protein, after which a + 1 ribosomal frameshift incorporates an additional 13 aa	Mitochondrially localized; inhibits RIG-I/MAVS signalling pathway to suppress innate immunity
2	PB1	∼757	non-spliced mRNA	Translated from AUG1	Component of RNA polymerase complex; responsible for RNA chain elongation
	PB1-N40	∼718		Translated from AUG1; produces an N-terminal truncated protein	Function unknown; may regulate viral polymerase activity, affecting replication efficiency
	PB1-F2	57∼101		Translated from AUG1; produces a truncated protein	Induces mitochondrial apoptosis; clears immune cells; inhibits MAVS protein to suppress host antiviral immunity
3	PA	∼716		Translated from AUG1	Component of RNA polymerase complex; possesses endonuclease activity; involved in viral RNA synthesis and processing
	PA-X	∼252		Translated from AUG1; a + 1 ribosomal frameshift at the UUU CGU C motif yields a fusion protein comprising the N-terminal 191 aa of PA and a C-terminal domain encoded by the X-ORF	Suppresses host mRNA, inhibits host gene expression, weakens host immune response
	PA-N155	∼562		Translated from AUG1; produces an N-terminal truncated protein	Function unknown; may be related to viral replication regulation
	PA-N182	∼535		Translated from AUG1; produces an N-terminal truncated protein	Function unknown; may be related to viral replication regulation
4	HA	∼566		Translated from AUG1	Mediates viral attachment to host cells and membrane fusion; major neutralizing antigen
5	NP	∼498		Translated from AUG1	Component of RNA polymerase complex; important cross-reactive antigen
	eNP	∼504		Translated from an upstream alternative AUG initiation codon, generating an NP isoform with a 6 aa N-terminal extension.	First detected in 2009 H1N1 pandemic strain; increases H1N1 pathogenicity
6	NA	∼454		Translated from AUG1	Cleaves sialic acid residues; promotes virus release
	NA43	∼440		Translated from AUG2; miss entire cytoplasmic tail domain and part of transmembrane domain (−14 aa)	Function unknown, but may localize to viral membrane
7	M1	∼252		Translated from AUG1	Matrix protein; maintains viral structure; major structural protein
	M2	∼97	alternatively spliced mRNA	Translated from AUG1; splicing induces a + 1 frameshift after codon 10, allowing translation to continue in the +1 frameshift to produce the full M2 protein	Ion channel protein; regulates intraviral pH; facilitates viral uncoating
	M42	∼99		Translated from AUG2	Similar function to M2; only found in some influenza strains
8	NS1	∼230	non-spliced mRNA	Translated from AUG1	Nonstructural protein; antagonizes host antiviral response
	NS2 (NEP)	∼121	alternatively spliced mRNA	Shares the AUG1 start codon and first 9 codons with NS1; after splicing, translation continues in an alternative open reading frame to generate the C-terminal domain	Nuclear export protein; mediates RNP export from nucleus to cytoplasm
	NS3	∼187		Translation from AUG1; an A374G nucleotide substitution generates a novel splice variant lacking 126–168 aa.	Rare splice variant; may regulate host adaptation and cross-species transmission barrier
	NEG8 (NSP)	∼167		Unclear; translation may initiate from a negative-sense open reading frame	Function unknown
	tNS1	∼150		Translated from AUG5 or AUG6	Truncated NS1 protein; inhibits IRF3

aa: amino acid

AUG: initiation codon

## The low mutation tolerance of M1

The surface glycoprotein genes of IAV, *HA* and *NA*, have a high degree of mutation tolerance, essential for evading the host immune system, while preserving conserved regions critical for their structural and functional integrity [10]. In contrast, the internal genes of IAV evolve more slowly, with *M1* being among the slowest evolving [11]. For example, the *M* gene of human seasonal influenza viruses evolves 5–10 times slower than the *HA* gene, while the *M2* gene evolves even faster than the *M1* gene. The M1 proteins of IAVs derived from both avian and mammalian hosts, including diverse subtype combinations of H1-H13 and N1-N9, exhibit over 95% amino acid sequence identity [11, 12]. The high degree of sequence conservation of M1, potentially due to lower selective pressure, obviates the necessity for frequent antigenic shifts. More significantly, M1 engages in interactions with numerous viral and host factors, thereby playing a pivotal role in the viral life cycle. The limited diversity of M1 is ascribed to its multifaceted functions, which may make it unable to accommodate excessive mutations [13]. Notably, the amino acid residues at positions 7, 8, 9, 18, 19, and 20 of M1 are exceptionally conserved; any substitutions at these sites can lead to a 10-100-fold reduction in virus titre [14]. The nuclear localization signal (NLS) of M1, specifically residues ^101^RKLKR^105^, prefers the native amino acids, with only positions 101 and 105 being permissive to mutations [15]. Furthermore, it has been indicated that the nuclear entry of M1 is facilitated by the highly conserved sequence ^163^RQMV^166^ [16]. Additionally, residues C148, C151, H159, and H162, which are instrumental in detecting pH changes during the uncoating process of M1, are also notably conserved [17]. The low mutation tolerance of these residues further underscores the indispensable role of M1 in the viral life cycle.

## The structure of M1

**Figure 1.**
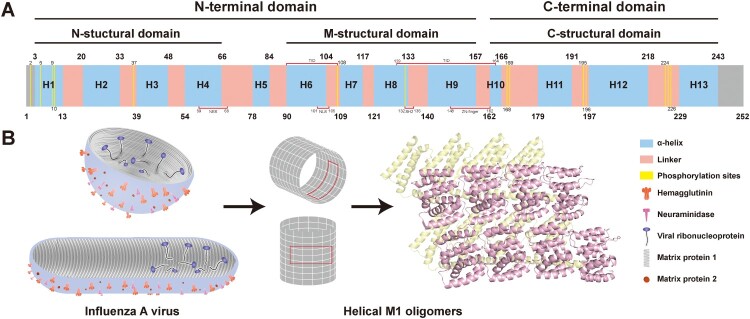
Structure of the influenza A virus M1 protein. A: M1 schematic diagram. This diagram highlights the N-terminal and C-terminal domains of M1, marks the positions of various α-helices (H1-H13) and linker regions, and indicates the locations of important functional regions and phosphorylation sites on M1. B: The left image in this panel shows cross-sections of spherical and filamentous IAV particles. The surface distribution of trimeric HA, tetrameric NA, and M2 is depicted. Beneath the viral envelope, tightly packed linear M1 polymers are shown, along with eight vRNPs encapsulated by the M1 protein. The middle image shows part of a monolayer of hollow helical M1 oligomers. The right image is an enlargement of the red-boxed area from the middle image, illustrating the interactions and arrangement of eight M1 proteins. In this figure, pink represents the N-terminal domain facing outward, while yellow represents the C-terminal domain facing inward.

The M1 protein, the predominant structural protein of IAV, forms a hollow cylindrical structure beneath the viral envelope. Its periodicity resembles a helical bead chain (Figure 1B) [18, 19]. Structurally, the protein contains two distinct domains: a compact N-terminal domain (NTD) and a conformationally flexible C-terminal domain (CTD) in solution. The X-ray crystallography at pH 4.0 and 7.0 has determined the relatively compact, globular structure of the N-terminal portion of M1 (residues 2-158). However, the lengthy and flexible CTD remains unsolved due to digestion during the crystallization process. The X-ray analysis showed that about 90% of the M1's secondary structure is composed of α-helices with three structural domains: the N structural domain (comprising α-helices H1-H4, residues 1-66), the M structural domain (H6-H9, residues 88-158), and the putative CTD (H10-H13, residues 167-252). The “L region” (residues 67-87), composed of L4-H5-L5, serves as a linker between the N and M domains at the N-terminus. A loop region, L9 (residues 162-166), bridges the M domain to the C domain at the C-terminus, with the conserved ^163^RQMV^166^ motif harbouring the Q-M cleavage site (Figure 1A) [20]. Peukes *et al.* [19] structurally characterized the M1 matrix layer in virus particles and virus-like particles (VLPs) assembled from HA, NA, M1, and M2. Results indicated that the M1 NTD, composed of nine α-helices, faces outward, engaging the plasma membrane (PM) with a positively charged patch spanning α-helices 5, 6, and 8. Conversely, the M1 CTD, folded as a trimeric coiled coil, is directed inward. It establishes contacts, via a trans interface, with the M1 NTD of neighbouring M1 monomers positioned away from the membrane. Furthermore, during viral budding, M1 exhibits distinct spatial orientation: 91% of viral particles display right-handed M1 helical arrangements (1-7 helices per turn), with the remainder adopting left-handed configurations. In right-handed arrangements, 80% of M1 chains orient their free NTDs toward the budding apex and free CTDs toward the base. The subenvelope M1 matrix layer forms a single helical layer. However, M1 molecules with antiparallel NTD-CTD orientations assemble into multilayered helical structures beneath the envelope [21]. Lisa *et al.* [22] successfully produced soluble full-length M1 protein in *vitro*, exposing two distinct M1 conformations. At neutral pH, full-length M1 adopts a highly ordered monolayer helical structure analogous to the matrix layer in intact viral particles. Under low-pH conditions, the M1 matrix layer collapses, detaches from the viral membrane, and forms more compact multilayer helical oligomers.

## The multifaceted functions of M1 in the viral life cycle

**Figure 2.**
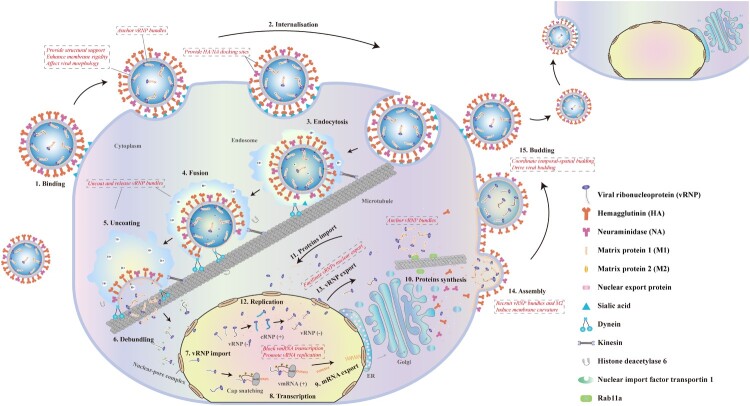
Life cycle of influenza A virus, highlighting the influence of the M1 protein on specific stages of virus replication. 1. The virus attaches to sialic acid-containing receptors on the cell surface via HA. 2. The virus is primarily internalized through clathrin-mediated endocytosis. 3. The formation of the endosome facilitates the entry of the virus into the cell. 4. The endosome moves toward the nucleus along microtubules, driven by dynein and myosin, while the acidification of the endosome triggers HA-mediated fusion between the endosomal and viral membranes [24]. 5. The histone deacetylase 6 promotes the disassembly of M1 and the vRNPs releases into the cytoplasm [27]. 6. The nuclear import factor transportin 1 facilitates the dissociation of M1 from the vRNPs, unwinding the vRNPs [28]. 7. vRNPs are transported into the nucleus via the classical importin pathway. 8. The heterotrimeric viral polymerase on the vRNPs captures the first 10-13 nucleotides from the capped host mRNA and uses them, along with the vRNA template, to transcribe viral mRNA. 9. The synthesized viral mRNA is exported to the cytoplasm for translation. 10. Viral proteins are synthesized in the endoplasmic reticulum and Golgi apparatus. 11. Newly synthesized viral proteins are imported into the nucleus to promote vRNP assembly. 12. The polymerase residing on the vRNPs replicates vRNA into cRNA, which binds to recently imported PB2, PB1, PA, and NP to form cRNP, leading to replication of progeny vRNPs. 13. Progeny vRNPs are exported to the cytoplasm with the help of M1 and NEP. 14. Viral transmembrane proteins, M1 protein, other viral proteins, and progeny vRNPs accumulate and assemble in lipid raft regions of the plasma membrane. 15. The viral transmembrane proteins induce budding, with the bud membrane incorporating into the developing virion. The cytoplasmic M1 oligomerizes to form the matrix layer beneath the membrane, while the genomic vRNP bundle is recruited to the tip of the budding virion [46, 47]. M2-mediated scission separates the viral membrane from the plasma membrane, and NA’s sialidase activity releases the newly formed viral particles from sialic acid-containing receptors on the cell surface [57].

M1 is synthesized during the late stages of virus infection and shuttles between the nucleus and cytoplasm, fulfilling multiple roles in the influenza virus life cycle, including uncoating, transcription, nuclear export of vRNP, and assembly and budding of virion (Figure 2).

### Uncoating to release vRNPs

The three-dimensional structure of the eight vRNP segments in IAV has shown that their arrangement within the virion is significantly supported by the M1 scaffold. Notably, this M1-vRNP complex forms and dissociates at different stages of the viral life cycle [23]. To start the viral replication, vRNPs must be released into the cytoplasm upon cellular entry, necessitating viral uncoating. This process, which entails the disassembly of the M1 matrix scaffold during the fusion of the viral envelope with the late endosomal membrane, is essential for vRNP release. Upon cell entry via endocytosis, V-ATPase on the endoplasmic reticulum (ER) membrane utilizes ATP hydrolysis to pump H^+^ and K^+^ from the cytoplasm into the ER lumen, modifying the ER’s pH. This induces a pH-dependent conformational change in HA, exposing the N-terminal fusion peptide of HA2, which then inserts into the ER membrane. Simultaneously, HA’s C-terminal anchors in the viral membrane, facilitating fusion between the viral and ER membranes [24, 25]. The low-pH environment activates the M2 proton channel, facilitating the transfer of H+ into the virus. This triggers a conformational change in the M1 protein, reducing its interaction with vRNPs and causing their asymmetric distribution. As the pH decreases further, the highly conserved, positively charged histidine cluster on the M1 matrix layer detects the H+ influx, prompting M1 protein dissociation. This results in the viral capsid opening and the M1 matrix layer disassembly [26]. The histone deacetylase 6 (HDAC6), a host factor, interacts with unanchored ubiquitin chains on the M1 protein, connecting it to dynein, dynactin, and myosin II. This interaction facilitates the disassembly of the M1 matrix shell, leading to the release of eight M1-vRNP complexes into the cytoplasm [27]. Following this, the nuclear import factor transportin1 (TNOP1) binds to the exposed PY-NLS on M1, thereby promoting the separation of M1 from vRNP [28].

### Timely blocking the transcription of viral mRNA

The proteins of IAV synthesize in the cytoplasm, while vRNA replicates within the nucleus. Upon synthesized, the PB2, PB1, PA, and NP proteins translocate to the nucleus to assemble the vRNP complex, consisting of eight vRNA segments. Blocking the nuclear export of vRNPs occurs when anti-M1 antibodies are introduced into infected cells or when the protein kinase inhibitor H7 inhibits M1 synthesis. These findings point to a necessity for M1 in the nuclear export of progeny vRNPs and indicate that M1 must enter the nucleus to assist in this process [29]. A canonical NLS enriched with basic amino acids, has been identified at the sequence ^101^RKLKR^105^ within M1's multifunctional motif (residues 91-105). The NLS forms an exposed loop on the surface of the N-terminus of M1, sandwiched between α-helices H6 and H7. The NLS is indispensable for M1's nuclear entry, with the host protein importin-α assisting this transfer by forming a heterodimer with the importin-β receptor upon NLS recognition [29]. Once in the nucleus, M1 swiftly suppresses viral mRNA transcription. As a non-glycosylated negative regulatory factor, M1 possesses two transcriptional inhibition domains located at residues 90-108 and 129-164, where residues R/K95, K98, and K102 can directly interact with vRNPs [30]. These interactions are critical for the inhibition of the RdRp, composed of PB2, PB1, and PA, thus preventing further transcription of viral mRNA. For example, amino acid substitutions targeting the NLS core region, exemplified by the R101S and R105S mutations in the A/WSN/1933 viral strain, disrupt the signalling function of this domain, thereby reducing the nuclear transport of the M1 protein [13]. Additionally, the nuclear import of the M1 protein is regulated by post-translational modifications, including phosphorylation at the Y132 residue. Phosphorylation at this site modulates the binding affinity between M1 and importin α1; its inhibition significantly reduces their interaction, thereby diminishing M1 nuclear transport efficiency [31].

### Facilitating the nuclear export of vRNPs

Although the M1's role in facilitating the nuclear export of vRNPs is well established, the precise mechanism remains elusive. Upon nuclear entry, M1 interacts with vRNPs through its C-terminus (residues 165-252) [26]. Previous studies have demonstrated that SUMOylation at the K242 residue within the M1 protein’s C-terminal structural domain modulates the interaction between M1 and vRNP complexes. Substitution of K242 with glutamic acid (K242E) markedly diminishes binding affinity between M1 and vRNPs, thereby disrupting the assembly efficiency of the M1-vRNP complex. This disruption subsequently impedes nuclear export of vRNPs from the host nucleus, ultimately inhibiting viral replication [32]. However, researches have shown that the M domain of M1 engages with vRNPs by binding to the C-terminus of the NP protein. For instance, vRNA or vRNP can bind to the NLS found in the M domain of M1 [33]. The trimeric complex NEP-M1-vRNP is formed when the NLS of M1's N-terminus binds to the C-terminus of NEP, in a sandwich-like structure [34]. The cellular nuclear export protein, chromosome maintenance 1 (CRM1), and its auxiliary factor Ran·GTP recognize the nuclear export signal (NES) at the N-terminus of NEP (residues ^12^ILMRMSKMQL^21^). This recognition enables CRM1 to bind to NEP in a Ran·GTP-dependent manner, further assembling into a larger nuclear export complex (CRM1-Ran·GTP)-NEP-M1-vRNP. Ultimately, this massive complex enters the cytoplasm through the nuclear pore, facilitated by the coordinated actions of CRM1, Ran, and NEP. Following nuclear export, the complex disassembles due to the hydrolysis of Ran·GTP, releasing the vRNP [35, 36]. The newly assembled vRNPs aggregate near the microtubule-organizing centre (MTOC), bind to Rab11a vesicles, and form vRNP bundles. These bundles are then transported to the cell membrane via vesicles along the MTOC’s microtubule network [37, 38]. This has led to the proposal of both the original model [39, 40] and the refined “daisy-chain” models [41]. Additionally, it should be emphasized that a portion of the newly synthesized M1 remains in the cytoplasm to prevent vRNP that has exported from the nucleus from returning to the nucleus [29, 40].

Other studies have shown that, similar to its role in nuclear entry, the NES (residues 59-68) of M1 is capable of mediating the nuclear export of vRNP in conjunction with NEP, thus eliminating the requirement for “daisy-chain” mediation [42]. Co-transfection of the NES-deficient M1/69-252 with NEP into 293 T cells revealed that this variant predominantly localized to the nucleus. Furthermore, NEP co-localized with NES-deficient M1/69-252 within the nucleus, yet was unable to facilitate the nuclear export of the NES-deficient M1, possibly due to the NEP-M1 complex obscuring NEP's NES. However, in the presence of vRNP, the interaction between M1 and vRNP alleviates the steric hindrance, thereby exposing NEP's NES. Consequently, the NEP-M1-vRNP complex facilitates the nuclear export of vRNP [42].

### Orchestrating viral assembly

M1 orchestrates the assembly and budding of IAV, earning its designation as the “adapter” for viral assembly. Following nuclear export, the NEP-M1-vRNP complex dissociates, enabling M1 to segregate and approach the cytoplasmic membrane. Studies have shown that viral particle assembly necessitates a threshold concentration of the M1 protein; insufficient M1 delays this process. To compensate, the virus optimizes M1's nucleocytoplasmic distribution and membrane localization via phosphorylation-dephosphorylation regulation [43]. However, the precise mechanisms governing M1's spatiotemporal regulation of these modifications remain elusive. IAV appears to employ early-stage phosphorylation to enhance M1 binding to the nuclear transport receptor importin-α, promoting M1 nuclear import and involvement in vRNP nuclear export, as illustrated by M1 Y132 phosphorylation [31]. Conversely, late-stage dephosphorylation may counteract PKG-mediated M1 degradation, enabling its plasma membrane accumulation, exemplified by M1 A37 dephosphorylation [43]. To date, potential M1 phosphorylation sites have been identified at positions 2, 5, 9, 10, 37, 108, 132, 168, 169, 195, 196, 224, 225, and 226 [31, 44, 45]. Subsequently, acting as a central hub, M1 induces membrane curvature by interacting with the PM via associations with the cytoplasmic tails of HA, NA, and/or M2 proteins and facilitates the integration of vRNPs into budding sites through its interactions with NP [46, 47]. The spatial organization among viral proteins within the PM has been delineated using immunogold staining techniques, revealing the lateral organization of IAV proteins at the budding site, which is also the location of protein assembly [48]. A key research focus is the mechanism by which M1 adheres strongly to the cytoplasmic membrane, despite lacking a membrane-targeting domain and any lipid modifications. The surface charge potential indicates a dichotomy in M1 monomer charge distribution, with one side being positively charged and the other negatively charged. The positively charged helices 5, 6, and 8 of the M1 NTD interact electrostatically with the negatively charged “lipid raft” region on the cytoplasmic side of the budding virion [19, 25, 49]. Studies have shown that M1's membrane targeting and attachment rely on the highly conserved arginine triplet R76/77/78, located within the positively charged L region at the N-terminus [50]. However, in uninfected cells, this interaction seems insufficient to establish a stable M1-cytoplasmic membrane linkage [51, 52].

During the viral assembly, M1 binding to the cytoplasmic membrane and its subsequent oligomerization are two closely intertwined processes. Upon membrane binding, M1 monomers or dimers induce membrane reorganization, forming helical oligomers that facilitate contact with unbound M1 molecules [18, 53]. The C-terminal residues 181-193, conserved across all IAV subtypes, are vital for M1 oligomerization [54]. Histidine residues in the M1's C-terminus have been implicated in M1 aggregation, potentially forming hydrogen bonds with S183 and/or T185 to promote oligomerization, and the double substitution (S183A + T185A) disrupts M1's quadruple symmetry, thereby aborting virus assembly [54]. Recent studies indicate that the reported tetrameric symmetry of M1 might reflect an experimental artifact. The relatively weak interactions between adjacent M1 monomers and the flexible linker connecting the M1 NTD and CTD enable the chains to slide past each other. This flexibility likely results in a dynamic, asymmetric oligomerization pattern of M1 [19, 21, 22]. A Recent study demonstrates that environmental shifts occurring throughout the entire IAV life cycle can dynamically and reversibly transition virions between filamentous and spherical morphologies [55]. This morphological plasticity implies that, during assembly, the M1 protein may both shape and adapt to the final virion architecture by modulating its oligomerization and enabling inter-monomer sliding.

### Mediating viral budding

In the study of the enveloped virus budding mechanisms have shown that endosomal sorting complex required for transport (ESCRT), crucial for multivesicular body formation in eukaryotic cells, is used by most enveloped viruses, including HIV, to complete viral particle budding from the host cell membrane [56]. However, IAV employs a unique evolutionary strategy by utilizing its own M2 ion channel protein for membrane scission independent of the ESCRT pathway, unlike other enveloped viruses that rely on the host ESCRT system [57]. IAV budding involves four stages: budding preparation, initiation, growth, and termination. Upon entering the cytoplasm and then reaching the PM, M1 induces asymmetry of the lipid bilayer by tightly interacting with the inner leaflet of the membrane [58]. Simultaneously, M1 attracts vRNPs to the viral membrane proteins embedded within the PM, leading to the aggregation of freely diffusing viral transmembrane proteins around the vRNP, thereby creating a dense cluster on the cell membrane [46, 59]. Once budding commences, M1 may drive filamentous virus elongation by polymerizing to overcome membrane tension and rigidity [21]. Subsequently, the M2 protein, located at the bud neck, leverages its amphipathic helix to induce positive membrane bending in a cholesterol-dependent manner. This localized alteration in membrane curvature facilitates the spontaneous scission of the bud membrane neck [57].

VLPs, which lack vRNPs and can disclose the minimal conditions for virus budding, are an excellent model for studying the budding process and mechanism of IAV. The specific role of M1 in the virus budding process remains a subject of debate, though. There are at least six distinct perspectives: (1) M1 alone is sufficient for virion budding and can form VLPs independently [60]; (2) M1 cannot form VLPs on its own and requires exogenous membrane-targeting sequences (e.g. fused to its N-terminus) or co-expression with viral membrane proteins to facilitate this process [52]; (3) Insufficient membrane targeting has led some researchers to propose that co-expression of HA, NA, M2, and M1 or HA (or NA) with M1 and M2 is necessary for VLPs formation [52, 61]; (4) As HA and NA can form VLPs independently, they are important for virus assembly and budding, with M1 being less significant [62]; (5) While HA, NA, and M2 support VLP formation, the resulting virions do not always exhibit consistent morphology with authentic virions, necessitating further research into their roles in VLP formation [63]; (6) True VLPs are only produced when the M protein is co-expressed with HA and/or NA, whereas expression of HA and/or NA alone primarily results in non-specific membrane vesicles [58].

The discrepancies among studies on the mechanisms of M1 membrane targeting and VLP assembly may arise from intrinsic differences in experimental systems and the complexity of protein regulatory networks. Divergent lipidomes, membrane properties, and host factor expression in 293 T, MDCK, or insect cell systems could influence the efficiency of M1 membrane localization. Additionally, post-translational modifications including palmitoylation and phosphorylation may modulate M1’s membrane affinity and plasma membrane accumulation, thereby affecting VLP assembly. Differences in the sensitivity and specificity of detection methods, including Western blotting and electron microscopy, may further contribute to inconsistent results. Other factors, including viral protein subtypes, culture duration, transfection efficiency, and buffer composition, may also indirectly influence VLP formation and detection, ultimately leading to divergent findings across studies. The above points suggest that each viral protein has a specific role in initiating viral budding: HA/NA facilitate membrane localization and curvature initiation, M1 serves as a scaffold connecting surface proteins to vRNPs, and M2 completes membrane scission and release. However, no single viral component can be solely attributed to this process. Therefore, further studies are needed to fully understand the mechanism of M1 in the formation of VLPs.

## M1 maintains viral morphology

IAV particles are pleomorphic, with spherical virions having a diameter of 80-120 nm and filamentous virions with a length of several microns. The conventional view has long held that clinical isolates are predominantly filamentous, whereas laboratory-adapted strains like A/Puerto Rico/8/1934 (H1N1) (PR8) and A/WSN/1933 (H1N1) (WSN) are typically spherical or elliptical [64, 65]. Extensive research has underscored the pivotal role of the M1 protein in maintaining and regulating viral morphology. For example, introducing the *M1* gene from the filamentous Udorn strain into the spherical WSN strain produces recombinant virions with filamentous morphology [66]. Similarly, the spherical PR8 virus can be transformed into a filamentous form by incorporating the *M1* gene from the 2009 pandemic H1N1 influenza virus, which predominantly exhibits a filamentous phenotype [67]. Further studies have identified that specific amino acid residues at both the N-terminus (positions 30, 41, 76, 77, 78, 87, 92, 95, 98, 101, 102, and 157) and C-terminus (positions 169, 183, 185, 198, 204, 207, 209, 215, and 218) of M1 are critically associated with virion morphology [54, 66]. For instance, the H5N1 virus A/Duck/Guangxi/53/2002 adopts a filamentous rather than spherical morphology when substitutions such as N30D and T215A are introduced. Similarly, the WSN strain develops filamentous virions when carrying M1 substitutions including K95A + K98A + R101A + K102A or K95R + T218A + V41A [68, 69]. Likewise, serial passage of the laboratory-adapted PR8 strain in guinea pigs led to filamentous phenotypes driven by M1 substitutions at residues 87, 92, 101, and 157 [65]. By contrast, mutations such as M1/R95K + E204D or M1/A41 V attenuates the filamentous phenotype [66].

However, recent research by Partlow *et al*. has overturned this this long-standing paradigm, revealing the remarkable morphological plasticity of IAV. When IAV experiences changes in extracellular or intracellular conditions, it adopts a filamentous morphology to enhance environmental resistance and improve survival. Once the stress is alleviated, the virus reverts to a more resource-efficient spherical form. This rapid, efficient, and reversible shift in the spherical-to-filamentous ratio can even occur during viral assembly. The study further highlights that this environmentally adaptive morphological change is a conserved genetic trait of IAV, rather than a strain-specific phenotype as previously believed [55]. This dynamic morphological switching strategy greatly enhances the adaptability and evolutionary resilience of IAV enabling it to effectively respond to immune pressure, host switching, and other complex challenges, thereby facilitating rapid optimization of survival and transmission. Notably, the previously observed morphological changes induced by *M1* gene replacement or specific point mutations may reflect the underlying molecular mechanisms by which IAV adapts to genetic background or environmental pressures. Undoubtedly, the central role of M1 remains crucial: by modulating its oligomerization and the flexibility of its monomeric conformation, M1 likely provides the structural foundation for IAV’s dynamic morphological transitions. Future studies should systematically investigate how environmental stress integrates with each and every genetic variation – particularly those in all polymorphisms, mutations, and allelic variants across the entire *M1* gene – to collectively drive IAV’s morphological plasticity.

## M1 influences viral transmission, pathogenicity and evolution

An even more important discovery is that the morphology of IAV is related to its transmission efficiency. The general consensus is that filamentous virus found in nature spreads more efficiently [70]. Evidence of M1 on viral transmissibility surfaced following the 2009 A/H1N1 influenza pandemic. In the ferret model, the filamentous A/Netherlands/602/2009 (H1N1) (NL602) strain was capable of efficient transmission, unlike the spherical PR8 strain. Utilizing reverse genetics techniques, three segments encoding HA, NA, and M from NL602 were incorporated into the genetic background of PR8, resulting in a filamentous recombinant virus that exhibited increased NA activity and a transmission capability similar to NL602 in ferrets. Further investigation revealed that the altered morphology, elevated NA activity, and successful transmission of the recombinant virus were all attributed to the N87S and R101G substitutions on M1 [71]. It was revealed that the *M1* gene from the filamentous A/California/4/2009 (CA-04) strain can restore the transmissibility of PR8 in guinea pigs. Notably, CA-04 encodes an alanine (A) at position 41 of M1, while the PR8's M1 has a valine (V) at this position [67], further indicating the importance of the M1/41 position for effective viral transmission [72].

The pathogenicity of IAV is associated with the M1 protein. For instance, the M1-41 V determined growth and pathogenicity of chimeric H17 bat influenza virus in cells and in mice [73]. When A/HK/1/68 (H3N2) strain was passaged through mice, Brown *et al*. [74] discovered that 14 amino acid sites on M1 had mutated. Notably, the M1/T167A, D232N, and M2/D44N changes that happened on M1 and M2 might greatly increase the virus's pathogenicity to mice. Fan *et al*. [75] found that although two duck-origin H5N1 virus strains were lethal to chickens, only one was pathogenic to mice, with the M1/N30D (K)+T215A substitutions on M1 being crucial for H5N1 virus's pathogenicity. These findings underscore the importance of M1 in modulating the pathogenic potential of IAV.

The M1 protein also traces the evolutionary trajectory of IAV. Our previous analysis of the *HA* gene of H9N2 viruses circulating in Chinese poultry revealed that the dominant lineages in China include Y3, Y8, G3, G4, and the B1-B4 clades, with the B4 clade emerging as the overwhelmingly predominant branch in recent years. Correspondingly, the *M1* gene has diversified into five major distinct evolutionary patterns (M1_P1_–M1_P5_). Among them, the Y3, Y8, and G3 lineages were exclusively associated with M1_P1_; M1_P2_ was predominantly detected in the B1 and B2 clades; while M1_P3_, M1_P4_, and M1_P5_ were present in both the G4 clade and the B lineage, with the latter being dominant. Further studies suggest that the epidemiological advantage of the H9N2 virus is strongly associated with specific M1 patterns, especially M1_P5_, indicating that M1 evolution not only reflects the prevalence of H9N2 but also serves as a critical driver of its adaptive evolution [76].

## M1 as a potential antiviral target

Current anti-influenza virus inhibitors primarily target the surface glycoproteins and the polymerase complex of IAV. M2 ion channel inhibitors, including amantadine and rimantadine, were once widely used, as they disrupt viral uncoating by blocking proton influx into the virion. However, their clinical utility has declined substantially due to the rise of drug-resistant strains [5]. NA inhibitors, including oseltamivir, zanamivir, and peramivir, are designed to block the release of progeny virions by competitively binding to the NA active site. Among polymerase inhibitors, baloxavir targets the PA endonuclease subunit to inhibit viral transcription; favipiravir acts as a nucleoside analog that incorporates into nascent RNA and disrupts PB1-mediated RNA synthesis; and pimodivir inhibits the “cap-binding activity” of PB2, blocking the “cap-snatching” process essential for viral mRNA synthesis [77]. Despite their widespread clinical application, these antiviral agents continually face the challenge of rapidly evolving drug-resistant influenza variants.

In contrast, M1, the most abundant structural protein of IAV, serves as a central “adapter” in multiple essential stages of the viral life cycle, including maintaining virion structural integrity, mediating uncoating, facilitating vRNP nuclear export, and orchestrating assembly and budding. Moreover, its sequence is highly conserved across diverse IAV subtypes, positioning M1 as a theoretically promising target for broad-spectrum antivirals. Studies have shown that small molecules such as PHE can specifically bind M1 and inhibit its self-oligomerization, thereby disrupting viral assembly and exhibiting antiviral activity both *in vitro* and *in vivo* against multiple strains, including seasonal H1N1, H3N2, and H5N1 [78]. Despite this potential, no M1-targeted drug has yet advanced to clinical use. This delay may due to the protein’s functional complexity and structural dynamics. The conformations of its NTD and CTD domains are highly plastic and pH-dependent, while its oligomerization involves asymmetric and dynamic interaction networks. These characteristics pose significant challenges for the design of small-molecule inhibitors that can stably and specifically disrupt critical functional interfaces. Furthermore, unlike enzymatic targets such as NA or the polymerase complex, which contain well-defined catalytic pockets amenable to drug binding, M1 functions primarily as a structural and scaffolding protein with no conventional binding cavities. Current studies on M1 inhibitors remain largely restricted to *in vitro* systems, cell cultures, or embryonated chicken egg models, with insufficient assessment of pharmacodynamics, pharmacokinetics, and safety *in vivo*. The scarcity of clinical and preclinical data further hinders the validation of their therapeutic potential and the exclusion of possible toxic effects. Therefore, despite its strong theoretical advantages, the multifunctionality and highly dynamic nature of M1 present substantial barriers to its development as a clinically feasible antiviral target. Overcoming these challenges will be essential to fully unlock the therapeutic potential of M1 in influenza intervention.

## Conclusion

As the most abundant structural protein in IAV, M1 serves as the core scaffold maintaining virion integrity and functions as a key molecular switch regulating critical stages of the viral life cycle, including assembly, budding, and vRNP nuclear-cytoplasmic transport. While M1’s activity is known to directly influence viral fitness, many mechanisms remain unclear. The molecular determinants underlying the formation of monolayer or multilayer helical M1 oligomers, and their differential impact on viral fitness, remain to be elucidated. It is imperative to dissect every biophysical pathway through which M1 senses extracellular stress-host protease gradients, immune pressure, pH fluctuations, redox shifts, nutrient depletion, temperature changes, mechanical forces, cytokine milieus, and any other environmental perturbation – and converts these diverse cues into rapid, cycle-to-cycle modulation of virion morphology. Additionally, given M1’s low mutational tolerance, how do interactions between its conserved functional domains and compensatory mutations in other viral proteins influence the global evolutionary trajectory of influenza viruses? The finding that M1 reflects the evolutionary trajectories of IAV underscores the value of studying its dynamic evolution for predicting influenza pandemic trends, refining vaccine design, and advancing novel antiviral strategies. It also underscores the imperative to integrate structural biology, molecular interaction analysis, biophysics, computational modelling, and every other relevant discipline to systematically dissect the conformational dynamics of M1 across viral life cycle stages. Importantly, research on M1 provides a theoretical basis for developing antiviral agents targeting viral life cycle and reveals new dimensions for exploring how IAV adapts evolutionarily under host-selective pressures.

In conclusion, M1 is a cornerstone of IAV biology, bridging structural integrity with functional diversity. Research on M1 has not only advanced our understanding of influenza viral replication but also opened new avenues for addressing the most persistent and ever-evolving influenza threats to global health. As we continue to unravel the complexities of M1, we move closer to a more comprehensive understanding of influenza virus biology and its implications for public health.
